# The Engineering Side of Hospital Work

**Published:** 1905-03-04

**Authors:** 


### HOSPITAL ADMINISTRATION.

### CONSTRUCTION AND ECONOMICS.

### THE ENGINEERING SIDE OF HOSPITAL WORK.

*Continued from page 376.*

### V.—REFUSE DESTRUCTORS AND DISINFECTORS.

THE principles upon which both of these apparatus are based are the same, the employment of heat to destroy germs and organic compounds that might lead to the propagation of disease; but whereas in the destructor, called the incinerator where it is used in hospitals, the object is to utterly destroy all the substance that is presented to the apparatus, in the case of the disinfector the object is to remove the germs that may be present in the pores of the linen, etc., in such a manner that it may be used again. One of the difficulties in connection with both forms of apparatus is the great tenacity to life possessed by the germs, and the resistance offered by the organic compounds against which the apparatus are directed. In the case of refuse destructors, the organic compounds require a temperature of at least 1,500° Fahrenheit, and it is usual to provide 2,000°, and the reason is, the earlier forms of destructors, arranged to get rid of the ordinary refuse of towns, so that the nuisance which had existed previously should be done away with, were found to be more of a nuisance in their immediate neighbourhood than the systems they displaced. In the earlier destructors the refuse was burnt, and it was assumed that burning simply was sufficient for all purposes, the temperatures being those of the ordinary boiler furnace, and sometimes less, from 400° to 600° F., or thereabouts. But at this temperature the organic compounds referred to escaped into the surrounding atmosphere, from the destructor chimney, the nuisance being accentuated by the foul smells sometimes emitted by the escaping gases at the chimney top. The trouble was also accentuated by another mistake that was made in those days. The gases produced by the burning refuse were given a considerable velocity in escaping, so as to create a good draught in the furnace where they were being formed, and as with similar conditions in the burning of coal the furnaces of steam boilers, the high velocity of the draught carried some of the dust from the refuse into the chimney, with the gases, and deposited them on the surrounding objects.

### THE MODERN PROCESS OF REFUSE DESTRUCTION.

Strictly speaking the modern refuse destructor consists of three parts, performing three distinct operations, that of drying the refuse, that of burning proper, and that of cremating the products of combustion of the furnace, raising them to the temperatures mentioned above. It is usual to arrange that the three operations are performed consecutively, and by the ordinary working of the apparatus, the three parts forming one compact apparatus. Apparatus intended for hospital use necessarily differ in some respects from those intended for ordinary town's refuse. The quantities to be dealt with are much smaller even in the largest hospital, to those in the case of even small towns. On the other hand, the substances to be dealt with are sometimes different. Town's refuse varies very largely from day to day, and often from that of hospitals. It consists very largely of vegetable matter, fish, and butcher's offal, while there is also usually a large percentage of such things as old boots, and other oddments. There are never any such things as come from the dissecting or post-mortem room. Further, there is nearly always a large percentage of cinders which add to the calorific value of the refuse. It should be understood that the modern town's refuse destructor is self-supporting in the matter of fuel. Every substance has some calorific value, and the average refuse has the ability to raise 1 lb. of water to steam, with the consumption of 1 lb. of the refuse, the ordinary standard for coal being 8 lb. of water raised to steam with the consumption of 1 lb. of coal, while some coals give as much as 12 lb. On the other hand some refuse gives as much as 2 lb. of steam raising power for every pound consumed, and again in some of the poorer continental towns the figure sinks to less than 0·5 lb.

The *modus operandi*, with the usual refuse destructor is as follows:—The refuse is collected in carts, and tipped, at the destructor, on to a drying hearth, forming usually part of the furnace itself, but not of the zone where actual combustion is taking place. The drying hearth receives heat from the furnace, and the object is to expel any moisture that may be present in the refuse, and there is often a great deal, before it is made to take part in actual combustion. A variation of this is, the refuse is tipped into hoppers in close proximity to the furnace, where it is dried, and it is fed by hand from the hopper, whose mouth is near the furnace door, into the furnace. In any case, after the refuse is dried, as far as possible, it is drawn on to the bars which form the furnace grate, where it comes into contact with refuse already in process of combustion. The process is very similar to that of adding coal to an ordinary boiler fire, except that greater precautions are taken not to allow the ingress of cold air and not to allow of the escape of the green gases, as they are termed. From the furnace proper the products of combustion pass through a flue, specially arranged to be maintained at a very high temperature, the flue being lined with fire bricks of a very refractory nature, and the bricks being maintained at white-heat. The result of this is, the products of combustion are subjected to the very high temperatures mentioned, and all organic compounds are thoroughly broken up. Special arrangements are also made for preventing the dust that is formed in rather large quantities, with some kinds of refuse, from passing to the chimney. In one form of destructor the dust passes through a whirling chamber on its way to the chimney, the dust settling in pockets on the outside of the chamber, and in another it passes through what is practically an expansion chamber, where the velocity of the gases is suddenly reduced, and the dust caught. It is also usual to heat the air with which the destructor furnace is fed, by means of some of the heat left in the hot gases after they have passed through the cremating flue. It will be understood that in many of these processes, where heat is employed and where substances are raised to a high temperature, to be afterwards lowered, it is a great advantage if the regenerative principle can be employed, some of the heat present in the substance that is to be cooled being delivered to some part of the substance that has to be heated, or to some substance that is to take part in the operation of heating. In all furnace work it is of very great advantage to heat the air with which the furnace is fed, because the heat necessary to raise its temperature to that of the other substances on the furnace bars is then not taken from the substances in process of combustion themselves, nor from the hot gases in formation. Where cold air is admitted to a boiler or other furnace the efficiency of the apparatus is considerably reduced by the above, and by the partial stoppage of the processes of combustion. Where the heat that is to be delivered to the air that is to be fed to the furnace can be taken from the hot gases that should be cooled before they are allowed to pass into the outer atmosphere, the gain is great. This is accomplished in various ways, the principle being the same in all; the hot gases are made to pass through or round tubes, or other vessels, in which, or round which, the air for the furnace is passing.

In the modern refuse destructor, the hot gases from the furnace, after they have been thoroughly cremated, are taken through the flues of a boiler, and caused to generate steam for any purpose that may be available, such as the generation of electricity for electric light, or power.

It will be seen in the next article how the hospital incinerator has been adapted from the town's refuse destructor to hospital requirements.

### Disinfecting.

As indicated above, disinfection is a different process to destruction, inasmuch as it is intended to destroy the poisonous germs, without destroying the fabrics in which they may have harboured. There are not many disinfecting apparatus on the market, but, as usual, all are constructed on certain main principles. It will be understood that the germs in question are embedded in the fabrics to be treated, and one of the difficulties of the problem is that of penetrating to the interstices in which they lie, killing them, and washing, or otherwise clearing them out, without damaging the fabric. Hot air will do the work, but it takes longer than steam. When the clothes, etc., are put into an apparatus intended to kill and drive out the germs, the pores of the fabrics contain air as well as germs, and hot air under pressure can be made to pass everywhere, into all the pores, killing all the germs and passing out with them. Steam, however, has certain advantages over hot air. Steam is a very convenient means of storing and delivering heat to substances such as those under discussion, and, in fact, to a good many other substances. It is very easy, with modern appliances, to raise steam from water and to give the steam any temperature, and any other physical properties that may be desired. Steam will also give up its heat to other bodies very readily, and then only presents a very small quantity of substance to be got rid of, when it has assumed the liquid form. On the other hand, to use air it is necessary to deal with very much larger quantities to perform the same work, and air does not deliver its heat so readily to other objects as steam. The operation of air is complicated, in many instances, by how much moisture it contains, the heat that should have been devoted to raising the temperature of another body being sometimes devoted to converting the water it carries into vapour, not necessarily steam proper, but absorbing a large quantity of heat.

### The Different Conditions of Steam.

Steam may be arranged under a very wide variety of conditions, but for the purpose of this article three may be considered:—Saturated steam at atmospheric pressure; steam superheated at atmospheric pressure; saturated steam at pressures above that of the atmosphere.

Saturated steam is steam as it comes from the boiler. It is not pure steam, but contains watery vapour that is not steam proper, that has been brought over from the water in the boiler by the mechanical action of the steam itself upon small globules of water imprisoned in the steam. This watery vapour that is present in saturated steam forms one of the troubles of the steam engineer. It is continually condensing to water when it is not wanted to, and as continually being converted into either steam or vapour at the expense of heat that is required elsewhere.

Superheated steam is steam that has been heated after its connection with the boiler has been severed. The heat applied raises the watery vapour to proper steam, and increases the pressure, or the volume of the whole body of the steam.

Saturated steam at pressures above atmospheric pressure is steam that has been exposed to heat after steam has been generated, but without severance of the connection with the boiler. When heat is applied to a boiler in which there is water, first the water has its temperature raised, degree by degree, as the heat is absorbed, each B.Th.Unit, approximately, raising the temperature of each lb. of water one degree, till the critical temperature arrives at which steam is generated, 212° Fahr. at ordinary atmospheric pressure. At this point a very large absorption of heat takes place. The lb. of water that had absorbed only about one B.Th.Unit for each increase of one degree of temperature now absorbs 966 B.Th.Units without raising its temperature at all. But, in any boiler, as heat continues to be applied, and as more and more steam is generated, if the steam is not taken away to do work, but is confined within the steam space of the boiler, the steam becomes more and more confined, and its pressure rises, and so does its temperature. There is a definite temperature for every steam pressure. Hence it is possible to deliver steam to any object, at any temperature, by delivering it at any particular pressure, and this is what is meant by steam at pressures above the atmosphere. The practical point in this matter is that the temperature of the steam is higher and higher as its pressure is increased.

In the next article the writer will explain how the different properties described have been made use of in the construction and working of disinfecting apparatus.

